# *Notes from the Field: Veillonella* Misidentified as *Francisella tularensis* — Idaho, 2016

**DOI:** 10.15585/mmwr.mm6621a4

**Published:** 2017-06-02

**Authors:** Kris K. Carter, Erin M. Peterson, Robert L. Voermans, Kenneth S. Anderson, Tamara Cox, Ahmed M. Kassem, Christopher L. Ball, Christine G. Hahn

**Affiliations:** ^1^Career Epidemiology Field Officer Program, Office of Public Health Preparedness and Response, CDC; ^2^Idaho Division of Public Health; ^3^Eastern Idaho Public Health, Idaho Falls; ^4^Epidemic Intelligence Service, CDC.

In October 2016, the Idaho Bureau of Laboratories, Division of Public Health, was notified by hospital A’s clinical laboratory (a member of the Idaho Sentinel Laboratory Network) that a bacterial isolate cultured from a hospitalized patient’s knee joint fluid aspirate had been identified with 96% confidence as *Francisella tularensis* (a Tier 1 select agent*) by an in-house automated microbial identification system (AMIS). The isolate was submitted to the Idaho Bureau of Laboratories for confirmatory testing using Laboratory Response Network (LRN) reference methods. Hospital A laboratory personnel reported that the isolate had been manipulated on the open bench and certain laboratory workers had potentially been exposed. The Division of Public Health, hospital A, and Eastern Idaho Public Health initiated an investigation to confirm *F. tularensis*, assess potential laboratory exposures, and determine the source of infection. The investigation determined that the infectious agent was *Veillonella* and not *F. tularensis.*

The patient, a man aged >75 years, had a multiyear history of chronic unilateral knee pain, during which time he had received a series of three intra-articular injections of hyaluronate sodium 2 years previously, and several intra-articular injections of triamcinolone with bupivacaine, the last of which occurred 15 days before he sought care at hospital A for a swollen knee. Gram staining of an intra-articular aspirate obtained that day from the affected knee showed Gram-variable cocci. The aspirate was cultured under aerobic and anaerobic conditions. Slow-growing colonies of Gram-negative cocci were observed from the anaerobic culture, with limited growth in aerobic conditions. Because an anaerobic AMIS panel was not available, isolates from the aerobic culture were processed for identification and antimicrobial susceptibility on the AMIS using a panel specific for aerobic organisms. Identification of *F. tularensis* by the AMIS triggered notification of the Division of Public Health and revision of the patient’s antibiotic regimen from vancomycin, piperacillin, and tazobactam to ciprofloxacin. Eastern Idaho Public Health interviewed the patient and determined that he lived in a rural area and reported no recent exposure to potential sources of naturally occurring *F. tularensis* (e.g., ill animals, arthropod bites, contaminated water, or use of lawn mowers or string trimmers near dead animals).

The Idaho Bureau of Laboratories and Eastern Idaho Public Health provided CDC guidance on tularemia laboratory exposure (*1*) to hospital A and assisted staff members in assessing potential exposures of laboratory personnel. Among 24 employees interviewed by hospital A, 19 were considered to have potential exposure; all 19 elected to start antibiotic prophylaxis because of a high level of concern regarding risk and the approximately 48 hours required for confirmatory testing.

Using LRN real-time polymerase chain reaction methods, the Idaho Bureau of Laboratories tested the isolate for *F. tularensis* and *Brucella* spp.; no *F. tularensis* or *Brucella* spp. DNA was detected. Subsequent partial 16S ribosomal RNA (rRNA) gene sequencing identified a *Veillonella* sp. According to Clinical and Laboratory Standards Institute guidelines, *Veillonella* species-level identification could not be reported because the 16S rRNA gene fragment had a >99% sequence identity for both *V. parvula* and *V. dispar* without a <0.8% difference between them (*2*). Partial RNA polymerase subunit B gene (*rpoB*) sequencing conducted on the isolate found a >99% sequence identity to *V. parvula*, suggesting this as the most likely species of the isolate (*3*). After identification of *Veillonella* sp., the patient’s antibiotic regimen was changed to piperacillin, tazobactam, and ertapenem sodium; personnel who were receiving prophylactic antibiotics for potential *F. tularensis* exposure were informed that continuation of prophylaxis was not recommended or necessary. The Division of Public Health reviewed MedWatch data^†^ and did not identify any reports of *Veillonella* infection associated with hyaluronate sodium, triamcinolone, or bupivacaine. The source of this patient’s infection was not determined.

*Veillonella* spp. are small, slow-growing, nonmotile anaerobic Gram-negative cocci found as part of the normal flora of gastrointestinal, respiratory, and vaginal tracts. Although *Veillonella* spp. are classified as anaerobes, anaerobic organisms (including *Veillonella* spp.) have been observed growing in aerobic conditions for a limited time after isolation before becoming nonviable (*4*). Often considered contaminants of clinical specimen collection, *Veillonella* spp. have been rarely isolated from monomicrobial cultures of invasive infections. Predisposing factors for invasive infection have not been fully studied, but might include local or systemic immune suppression and localized anaerobic conditions produced by tissue necrosis, diminished blood supply, or prolonged infection with aerobes (*5*).

This is the first published report of misidentification of *Veillonella* sp. as *F. tularensis* by an AMIS and of isolation of *Veillonella* sp. from a nonprosthetic knee. Hospital and public health staff members responded appropriately to the preliminary misidentification of the isolate as *F. tularensis*; however, accurate identification would have obviated the need for time-intensive response activities, use of prophylactic antibiotics by hospital staff members, and inappropriately targeted antibiotic therapy for the patient. Clinical laboratories are advised not to use a commercial AMIS if a select agent is suspected in a clinical sample (*6*) and to consult with their LRN-biologic laboratory for guidance and sample referral. Clinicians should consider *Veillonella* spp. when receiving laboratory reports of *F. tularensis* generated by AMISs. Because *Veillonella* spp. are typically resistant to recommended or alternative antibiotic therapies for tularemia (i.e., streptomycin, gentamicin, tetracyclines, ciprofloxacin, and other fluoroquinolones), antibiotic coverage for both *Veillonella* spp. and *F. tularensis* could be considered until final microbial identification is available.

## References

[1] CDC. Tularemia fact sheet: managing potential laboratory exposures to *F. tularensis*. Atlanta, GA: US Department of Health and Human Services, CDC; 2015. https://www.cdc.gov/tularemia/resources/lab/tularemialabexposurefactsheet.pdf

[2] Clinical and Laboratory Standards Institute. Interpretive criteria for identification of bacteria and fungi by DNA target sequencing; approved guideline. CLSI document MM18-A. Wayne, PA: Clinical and Laboratory Standards Institute; 2008.

[3] Beighton D, Clark D, Hanakuka B, Gilbert S, Do T. The predominant cultivable *Veillonella* spp. of the tongue of healthy adults identified using *rpoB* sequencing. Oral Microbiol Immunol 2008;23:344–7. 10.1111/j.1399-302X.2007.00424.x 18582335

[4] Tally FP, Stewart PR, Sutter VL, Rosenblatt JE. Oxygen tolerance of fresh clinical anaerobic bacteria. J Clin Microbiol 1975;1:161–4. 1176601 10.1128/jcm.1.2.161-164.1975 PMC275000

[5] Brook I. Spectrum and treatment of anaerobic infections. J Infect Chemother 2016;22:1–13. 10.1016/j.jiac.2015.10.010 26620376

[6] Snyder JW, ed. General introduction, recommendations and biochemical procedures. In: American Society for Microbiology. Laboratory response network (LRN) sentinel level clinical laboratory protocols for suspected biological threat agents and emerging infectious diseases. Washington, DC: American Society for Microbiology; 2016. https://www.asm.org/images/PSAB/LRN/Intro316.pdf

